# Adverse Drug Reactions Related Hospital Admissions in Persons Aged 60 Years and over, The Netherlands, 1981–2007: Less Rapid Increase, Different Drugs

**DOI:** 10.1371/journal.pone.0013977

**Published:** 2010-11-12

**Authors:** Klaas A. Hartholt, Nathalie van der Velde, Caspar W. N. Looman, Martien J. M. Panneman, Ed F. van Beeck, Peter Patka, Tischa J. M. van der Cammen

**Affiliations:** 1 Section of Geriatric Medicine, Department of Internal Medicine, Erasmus MC, University Medical Center, Rotterdam, The Netherlands; 2 Department of Surgery-Traumatology, Erasmus MC, University Medical Center, Rotterdam, The Netherlands; 3 Department of Public Health, Erasmus MC, University Medical Center, Rotterdam, The Netherlands, Erasmus MC, University Medical Center, Rotterdam, The Netherlands; 4 Consumer and Safety Institute, Amsterdam, The Netherlands; Helmholtz Zentrum München, Germany

## Abstract

**Background:**

Epidemiologic information on time trends of Adverse Drug Reactions (ADR) and ADR-related hospitalizations is scarce. Over time, pharmacotherapy has become increasingly complex. Because of raised awareness of ADR, a decrease in ADR might be expected. The aim of this study was to determine trends in ADR-related hospitalizations in the older Dutch population.

**Methodology and Principal Findings:**

Secular trend analysis of ADR-related hospital admissions in patients ≥60 years between 1981 and 2007, using the National Hospital Discharge Registry of the Netherlands. Numbers, age-specific and age-adjusted incidence rates (per 10,000 persons) of ADR-related hospital admissions were used as outcome measures in each year of the study. Between 1981 and 2007, ADR-related hospital admissions in persons ≥60 years increased by 143%. The overall standardized incidence rate increased from 23.3 to 38.3 per 10,000 older persons. The increase was larger in males than in females. Since 1997, the increase in incidence rates of ADR-related hospitalizations flattened (percentage annual change 0.65%), compared to the period 1981–1996 (percentage annual change 2.56%).

**Conclusion/Significance:**

ADR-related hospital admissions in older persons have shown a rapidly increasing trend in the Netherlands over the last three decades with a temporization since 1997. Although an encouraging flattening in the increasing trend of ADR-related admissions was found around 1997, the incidence is still rising, which warrants sustained attention to this problem.

## Introduction

Medication use among older persons has grown over the last decades.[1] Pharmacotherapy has become increasingly complex[2] due to growing knowledge about disease pathophysiology, discovery of new drug therapies over time, and secondary preventive therapies, usually laid down in guidelines or protocols.[3] In 2007, persons aged ≥65 years constituted 14.4% of the Dutch population, whereas 44% of all drug prescriptions was for this age-group.[4] This is not surprising, as older persons in general have the highest prevalence of chronic and multiple (co-morbid) diseases. However, benefits of medication use are always accompanied by potential harm. Even when medication is prescribed in the recommended doses according to the guidelines,[3] Adverse Drug Reactions (ADR) can occur. The frequency of ADR increases with increasing age.[5]–[8], [9] Older adults are at increased risk of developing an ADR[10] due to their social setting[11], [12], polypharmacy[3], [11]–[15], co-morbidity[16], cognitive impairment[12], [15], and physiological changes affecting the pharmacokinetics and pharmacodynamics of many drugs.[2], [11], [17], [18]

Recent studies have shown that ADR-related hospital admissions are increasing,[8], [19] and account for approximately 5–12% of all hospital admissions in older patients[6], [7], [9], [11], [14], [20], [21] with a high in-hospital mortality rate of 8–10%.[9] Furthermore, ADR-related hospital admissions appear to be preventable in two fifth of cases.[11], [13], [20] All in all, ADR in the older population form a large public healthcare problem, resulting in significant morbidity, healthcare consumption and high costs. Because of ageing societies[22] and an increasing life expectancy[23], ADR might be expected to become even a more serious public health problem.

Since multiple studies on ADR-related hospital admissions in older persons and their possible preventability have been performed, awareness on ADR has increased.[24], [25] We were interested in time trends of ADR-related hospitalizations, and especially whether the increased awareness about ADR has led to an actual decline of ADR-related hospitalizations. However, there is a paucity of data on time trends in healthcare use due to ADR.[26] Therefore, the aim of this study was to provide accurate data on trends in ADR-related hospitalization in older patients over the last decades.

## Methods

Data on ADR-related hospital admissions were retrieved from Statistics Netherlands (CBS, The Hague, The Netherlands), which combines information of the National Medical Registration (LMR) and the National Hospital Discharge Registry.[27] The LMR collects hospital data of nearly all hospitals in the Netherlands. Data regarding hospital admissions, primary admission diagnosis (i.e., the most dominant reason for admission), gender and age are stored in this database. Data on hospital admissions, mortality, and population composition were verified with the official Birth-registry. The Birth-registry is used to identify individual patients in the National Medical Registration. Based on specific personal characteristics, such as date of birth, gender, and address it is possible to determine individual patients. A uniform classification and coding system by the LMR is used for all hospitals and has a high coverage (less than 5% missing between 1981–2005, 12.0% in 2007). The coding system did not change during the study period. Extrapolation to full national coverage for each year was done by Statistics Netherlands. An extrapolation factor was estimated by comparing the adherence population of the participating hospitals with the total Dutch population in each year of the study.[28] Demographic data were also collected from Statistics Netherlands. The mid-year population number was used as denominator in this study.

ADR were defined as: “Medicinal and Biological substances causing adverse effects in therapeutic use”, using the International Classification for Diseases, 9^th^ revision (ICD-9), code E930 – E949 (Table 1) throughout the study period. The E-codes of the ICD-9 classification are used to describe the external cause of injuries. Drug-classes used in this study were based on the ICD-9 codes (E930-E949). Official coding clerks register the diagnosis and injury mechanism of all hospital admissions, based on data obtained from medical records. For this study, hospital admissions in older patients with ADR as the primary admission diagnosis were collected over the period 1981–2007. Older persons were defined as persons aged ≥60 years. Numbers of ADR-related hospital admissions were specified for age and gender. Age-specific incidence rates, in 5-year age-groups, were calculated using the number of the ADR-related hospital admissions in that specific age-group, divided by the total mid-year population number within that specific age-group. The age-specific incidence rates were separated for both genders, and expressed per 10,000 persons of that specific age-group. Direct standardization, based on the mean population size per 5-year age-group throughout the study period, was used to calculate the overall age-adjusted incidence in males and females. Growth in the number of hospital admissions was calculated in percentual increases compared to the year 1981. This model has been used in a previous study.[29]

**Table 1:** International Codes of Diseases of the World Health Organization, 9^th^ revision, for Adverse Drug Reactions.

Code	Description
E930	Antibiotics causing adverse effects in therapeutic use
E931	Other anti-infectives causing adverse effects in therapeutic use
E932	Hormones and synthetic substitutes causing adverse effects in therapeutic use (including a.o. cortical steroids, androgens, ovarian hormones, insulins, and thyroid derivates)
E933	Primarily systemic agents causing adverse effects in therapeutic use (including a.o. anti-neoplastic, immunosuppressive drugs, bisphosphonates, vitamins and enzymes)
E934	Agents primarily affecting blood constituents causing adverse effects in therapeutic use (including a.o. anti-coagulants, anti-coagulant antagonists, anti-anemic agents, iron)
E935	Analgesics antipyretics and anti-rheumatics causing adverse effects in therapeutic use
E936	Anticonvulsants and anti-parkinsonism drugs causing adverse effects in therapeutic use
E937	Sedatives and hypnotics causing adverse effects in therapeutic use
E938	Other central nervous system depressants and anesthetics causing adverse effects in therapeutic use
E939	Psychotropic agents causing adverse effects in therapeutic use
E940	Central nervous system stimulants causing adverse effects in therapeutic use
E941	Drugs primarily affecting the autonomic nervous system causing adverse effects in therapeutic use
E942	Agents primarily affecting the cardiovascular system causing adverse effects in therapeutic use
E943	Agents primarily affecting gastrointestinal system causing adverse effects in therapeutic use
E944	Water mineral and uric acid metabolism drugs causing adverse effects in therapeutic use
E945	Agents primarily acting on the smooth and skeletal muscles and respiratory system causing adverse effects in therapeutic use
E946	Agents primarily affecting skin and mucous membrane ophthalmological otorhinolaryngological and dental drugs causing adverse effects in therapeutic use
E947	Other and unspecified drugs and medicinal substances causing adverse effects in therapeutic use
E948	Bacterial vaccines causing adverse effects in therapeutic use
E949	Other vaccines and biological substances causing adverse effects in therapeutic use

### Statistical analysis

To model the trend in hospital admissions, a regression model with Poisson error and log link was built with log mid-year population size (per 5-year age-groups) of each year of the study as offset factor. A linear spline model, with age, year, gender, and population size was built to assess whether the annual growth changed over the study period for both genders. The parameter for calendar year, corrected for gender and age-group was transformed into Percentage Annual Change (PAC). Our spline function accommodated two piecewise linear fits, connected with one another at the best knot[30], which was estimated with ‘Joinpoint Regression Program’, Version 3.3.1. (Statistical Research and Applications Branch, National Cancer Institute, USA). This program decided where to place the knot and showed the necessity for assuming a spline instead of a simple linear model. The best knot, for males and females together, was found to be January 1, 1997. Therefore this knot was used for the analysis in both males and females. The analysis including splines yielded estimates of annual changes in admission rates within each period (1981–1996 and 1997–2007 respectively). Comparison of these two periods enabled us to detect and quantify changes in the secular trend in overall admission rates. A likelihood ratio test was performed to assess the significance of the spline over a single trend for the study period. Interactions of the spline for gender were added and tested to investigate differences in trends for genders. A Chi^2^-test was used to detect changes in drug-classes and admission diagnosis. Statistical analyses were performed using SPSS software (version 16.1.1). A p-value <0.05 was considered as statistically significant.

## Results

**Figure 1:**
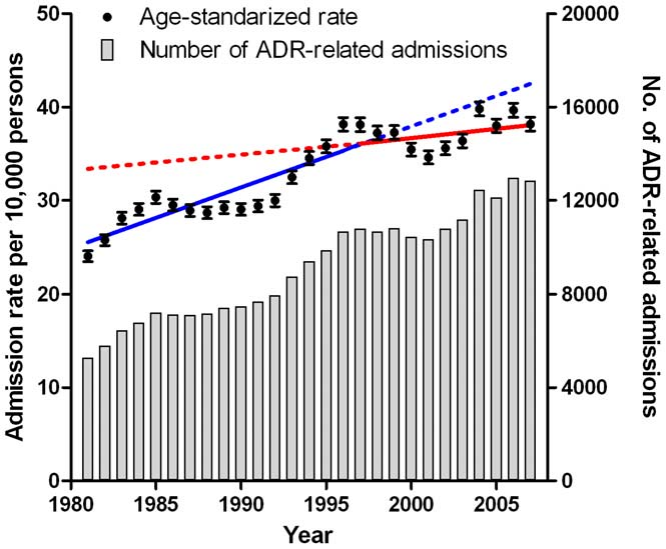
Adverse Drug Reactions, annual number and age-standardized rates per 10,000 persons aged ≥60 years in the Netherlands, 1981–2007. Separate regression lines are fitted to the period 1981–1996 (blue) and the period 1997–2007 (red). Solid lines indicate regression lines fitted to data points for the corresponding time period; dashed lines indicate the regression lines extrapolated for the remaining time period. Error bars indicate the 95% confidence interval.

During the 26 years of observation a total of 361,760 ADR-related hospitalizations were identified in the Netherlands (Table 2). Over two-thirds of the admissions (247,638) occurred in persons ≥60 years, which constituted only 17.6% on average of the Dutch population over 1981–2007. The total number of ADR-related hospital admissions in the Dutch population aged ≥60 years increased from 5,291 admissions in 1981 to 12,836 in 2007 (Figure 1). ADR-related hospitalizations increased by 175% in males aged ≥60 years (from 2,056 in 1981 to 5,651 in 2007) and by 112% in females aged ≥60 years (from 3,235 in 1981 to 7,185 in 2007). The overall standardized incidence rate (per 10,000 persons) of ADR-related hospital admissions in persons aged 60 years and older increased from 23.3 in 1981 to 38.3 in 2007 (Figure 1). The overall incidence rates (per 10,000 persons) increased in males from 21.3 in 1981 to 37.5 in 2007 and in females from 24.8 in 1981 to 39.0 in 2007.

**Table 2:** Overall numbers of Adverse Drug Reactions related hospital admissions in the Netherlands (1981–2007).

Years	0–59 years	%	60–69 years	%	70–79 years	%	80–89 years	%	≥90 years	%	Total	Female: Male ratio
1981–1985	17,913	36.2%	9,867	20.0%	13,239	26.8%	7,685	15.5%	742	1.5%	49,446	1∶0.69
1986–1990	17,524	32.5%	10,998	20.4%	14,969	27.8%	9,235	17.2%	1,122	2.1%	53,848	1∶0.74
1991–1995	19,400	30.8%	12,406	19.7%	18,064	28.6%	11,761	18.6%	1,434	2.3%	63,065	1∶0.78
1996–2000	23,095	30.2%	14,064	18.4%	22,321	29.2%	14,960	19.6%	2,050	2.7%	76,490	1∶0.72
2001–2005	25,278	30.8%	14,909	18.1%	22,641	27.5%	16,889	20.5%	2,473	3.0%	82,190	1∶0.75
2006–2007	10,912	29.7%	7,255	19.8%	9,771	26.6%	7,595	20.7%	1,188	3.2%	36,721	1∶0.75
Total	114,122	31.5%	69,499	19.2%	101,005	27.9%	68,125	18.8%	9,009	2.5%	361,760	1∶0.74

The overall annual growth of the incidence rate in the Dutch population over 60 years, corrected for age and population size was 1.78% (95% CI: 1.70–1.86) for males versus 1.47% (95% CI: 1.40–1.54) for females throughout the study period. A more detailed examination of the incidence curve of ADR (joint point regression analysis) revealed that the incidence growth has changed over time and can be divided in two phases: the incidence of hospital admissions due to an ADR increased strongly between 1981–1996 (2.56%, CI 95%: 2.46–2.67) and the percentage annual change slowed down between 1997–2007 (0.65%; CI 95%, 0.52–0.78). This flattening of the growth rate remained significant after correction for age and population size for both genders. Comparing 1986–1996 and 1997–2007, the annual growth rate decreased from 2.80% (95% CI: 2.63–2.96) to 0.38% (95% CI: 0.18–0.59) in males and from 1.86% (95% CI: 1.73–2.00) to 0.90% (95% CI: 0.72–1.08) in females respectively (p<0.001 for differences in slopes in both males and females).

Gender and age-specific incidence rates of ADR-related hospital admissions increased in all age-groups, both for males and females throughout the study period. All age-specific groups for both genders showed an increase in incidence of ADR-related hospital admissions when 2007 was compared to 1981. Among males (Table 3), the largest (relative and absolute) increase in incidence rates was seen in patients aged ≥90 years (162%, 95% CI: 80–283), the absolute increase in incidence rate was 43.3 per 10,000 persons (95% CI: 21.3–75.8). For females (Table 3), the largest increase was also seen in patients aged ≥90 years (112%, 95% CI: 66–169), the absolute increase was 37.2 per 10,000 persons (95% CI: 22.1–55.5).

**Table 3:** Age-specific incidence rates of Adverse Drug Reactions related admissions per 10,000 persons in the Netherlands.

Age	60–64 years		65–69 years		70–74 years		75–79 years		80–84 years		85–89 years		≥90 years	
	Males	Females	Males	Females	Males	Females	Males	Females	Males	Females	Males	Females	Males	Females
**1981**	12.8	11.3	17.9	16.8	24.3	24.4	30.3	35.8	34.8	48.1	38.1	49.2	26.8	33.4
**1985**	15.5	13.1	25.8	20.4	32.6	30.2	45.2	41.6	44.5	56.8	52.2	61.6	39.2	42.9
**1990**	15.4	12.2	23.4	19.7	34.0	29.0	46.0	38.6	47.2	49.2	54.5	54.5	39.1	45.1
**1995**	16.5	15.1	28.1	23.3	40.9	32.8	55.5	50.5	65.0	66.2	66.0	71.5	53.1	53.2
**2000**	15.9	15.5	26.7	23.4	40.6	31.9	55.9	48.4	69.7	62.4	69.8	71.7	48.9	62.2
**2005**	17.6	17.9	28.2	24.2	41.0	35.1	57.5	51.2	71.2	66.4	83.5	77.5	79.9	69.7
**2007**	17.7	19.9	27.0	26.8	42.7	33.4	62.5	48.3	71.0	64.8	72.0	78.7	70.2	70.6
Absolute Change* **(95% CI)**	5.0(2.9–7.3)	8.6(6.4–11.2)	9.1(6.2–12.4)	9.9(7.2–12.9)	18.4(14.0–23.2)	9.0(5.9–12.5)	32.3(25.6–39.8)	12.5(8.3–17.1)	36.2(26.9–47.0)	16.7(10.8–23.1)	33.9(20.7–49.9)	29.5(20.0–40.2)	43.4(21.3–75.8)	37.2(22.1–55.5)
**Relative** Change* **(95% CI)**	39%(23–57)	77%(57–99)	51%(35–69)	59%(43–77)	75%(58–96)	37%(24–51)	107%(85–132)	35%(23–48)	104%(77–135)	35%(22–48)	89%(54–131)	60%(41–82)	162%(80–283)	112%(66–169)

Abbreviation 95% CI, 95% Confidence Interval; * Change is 2007 compared to 1981.

**Figure 2:**
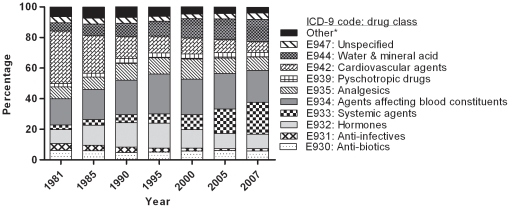
The composition of drug groups causing Adverse Drug Reactions related hospital admissions in persons aged ≥65 years in the Netherlands (1981–2007). The ICD-9 codes for Adverse Drug Reactions are shown. The main drug groups causing ADR-related admissions are shown separately in this figure. *“Other” drug groups were less frequently seen (<2% per group) and includes ICD-9 codes: E936-E338, E940, E941, E943, E945, E946, E948 and E949. The distribution changed during the study (p<0.001, *Chi* ^2^-test).

The distribution of medication groups causing ADR changed significantly (p<0.001) throughout the study period (Figure 2). The contribution of cardiovascular agents (ICD-9: E942) to ADR-related hospitalizations decreased from 36.0% in 1981 to 8.3% in 2007. Drugs affecting water, mineral and uric acid metabolism increased (ICD-9: E944) from 5.4% to 15.4%, primarily systemic agents (ICD-9: E933) increased from 3.2% to 24.4%, and agents affecting blood constituents (ICD-9: E934) increased from 17.9% to 24.2% between 1981 and 2007.

Approximately 45% of all ADR-related admissions were caused by six diagnostic groups (bleeding, gastrointestinal symptoms, anemia, cardiac symptoms, pulmonary symptoms, other) between 1991 and 2007 (Table 4). The distribution of admission diagnoses shifted during the study period (p<0.001).

**Table 4:** Primary admission diagnosis for Adverse Drug Reactions related hospital admissions in patients ≥60 years in the Netherlands (1991–2007).

	1991	1994	1997	2000	2003	2006	2007
**Bleeding**	**640**	**857**	**1,006**	**920**	**917**	**1,013**	**853**
**Gastrointestinal symptoms**	**349**	**442**	**446**	**491**	**527**	**549**	**513**
**Anemia**	**243**	**353**	**419**	**389**	**432**	**398**	**352**
**Cardiac system**	**398**	**513**	**564**	**452**	**433**	**435**	**377**
**Pulmonary system**	**123**	**191**	**290**	**329**	**390**	**474**	**486**
**Other**	**1,379**	**1,686**	**1,794**	**2,113**	**2,169**	**2,514**	**2,516**
Hypoglycemic coma	617	692	456	384	399	359	332
Dehydration/Electrolyte Disturbance	183	239	381	497	637	791	721
Urinary Tract Infection	109	136	168	133	170	239	283
Bone/Cartilage disease	70	94	115	313	214	201	202
Agranulocytosis	58	97	142	191	198	317	364
Obstipation	174	232	306	355	355	394	433
Pulmonary embolism	168	196	226	240	196	213	181

Data on primary admission diagnoses were available since 1991. Chi^2^-test for change in distribution of primary admission diagnosis was p<0.001.

## Discussion

This study shows that both the absolute numbers and the incidence rates of ADR-related hospitalizations in persons aged ≥60 years in the Netherlands increased strongly between 1981 and 2007. Although a slow down of the curve occurred in 1997, the incidence rates since then are still increasing, albeit at a less rapid rate. Of all ADR-related admissions, two-thirds were in the age-group of 60 years and older. The increase occurred in both males and females, although it was more pronounced in males and in the higher age-groups. Drugs classes leading to ADR-related hospitalizations shifted throughout the study period.

Our data represent an important first step in secular trend analysis of ADR-related admissions in developed countries. As far as we are aware from the literature, this study is the first to show a deceleration in the increasing incidence rates of ADR-related hospital admissions. Several factors may have contributed to this finding. The deceleration in growth of ADR-related hospital admissions started in the mid-nineties, after the introduction and widespread use of personal computers and software, with prescribing applications for doctors and pharmacists, which warns for possible drug interactions and errors.[31], [32], [33] Furthermore, due to professional publications and increasing media coverage, since the mid-nineties awareness about ADR among both professionals and the general public may have increased.[24], [25] Also standardized protocols and prescribing guidelines may have contributed to this trend.[3] Other studies did not show the decline in ADR-related hospitalizations, maybe due to their shorter study period[8] or ending in 2002, before flattening of the incidence rates of ADR-related hospital admissions had taken its full effect.[19] Theoretically the slowdown in ADR-related hospitalizations might also be (partly) caused by changes in admission policy at the Emergency Departments. However, a survey among Emergency Departments in the Netherlands[34] showed that the proportion of patients presenting with an ADR, followed by subsequent hospital admission, remained stable at 72% in the Netherlands between 1998 and 2008.

A major strength of this study was the availability of very accurate in-hospital data over an extensive period of 26 years (1981–2007) with almost complete national coverage. Absolute numbers of ADR-related hospital admissions in the Netherlands were recorded in a computerized database, with the same coding system (ICD-9) throughout the study period. This allowed us to gather reliable population-based data for our trend analysis. However, the data are only accurate within the limitations of the coding system, which is likely to be dependent on the accuracy of the data in the medical records and the recognition of ADR in the first instance by the patient's physician writing the record.

A number of limitations may have affected the interpretation of our findings. First of all, diagnosis codes were taken from a linked administrative database, which may be prone to coding errors and variation.[35] However, a recent quality survey showed a high accuracy of coded injury data (correctly coded in 91% of cases and in 9% incomplete).[36] This provides support for the validity of our data on ADR-related hospital admissions as extracted from the LMR database, and is comparable to the registration in New Zealand (period 1996–98).[37]

A second but unsolved limitation, however, is that the database does not contain information regarding specific drug(s), medication compliance, number of medications, co-morbidities, and clinical details of the ADR. Therefore, it was not possible in this study to draw conclusions regarding ADR details for specific drugs and certain high risk groups, for example patients with polypharmacy. Also, the database does not contain definitions of the ADR according to the Naranjo[38] or other algorithms, so a distinction between definite, probable, or possible ADR can not be made.

Third, we should take into account that underregistration of ADR might have occurred, since ADR recognition is very complex, especially in older adults, therefore ADR are not always noticed by medical personnel.[39], [40] For example, in older persons, falls and delirium caused by drug-use are still under-recognized as an ADR in current medical practice. For this reason, it is likely that the actual societal impact of all ADR-related morbidity, both mild and serious, exceeds the burden described in our study.[7]

Fourth, this study is based on the national situation in the Netherlands, with a full healthcare insurance coverage system and may be not representative for other countries. However, comparable increasing time trends were found in England[8] and Australia[19]. It therefore seems likely that the incidence of hospital admissions due to ADR will progress similarly in other developed countries.

In summary, drug prescription is a fundamental part of the care of older persons. Adverse drug reactions are a known drawback of medication use and represent an increasing public health problem, especially among older persons.[10] Changes in demographics alone cannot explain the increasing frequency of ADR and ADR-related healthcare demand in an ageing society. Although we found an encouraging decleration in the increasing trend of ADR-related hospitalizations around 1997, the incidence rates continued to increase from 1997 onwards, therefore our attention to this problem remains needed. Since ADR-related admissions have been shown to be preventable in two-fifth of the cases,[11], [13], [20] much is to be gained by further increasing the awareness among healthcare professionals that symptoms in older patients may be related to their drug use and by improving the ADR detection by the use of an ADR Risk score in daily practice.[41]
